# Neonatal Screening for Congenital Hypothyroidism with Focus on Developing an Indian Screening Programme

**DOI:** 10.17925/EE.2016.12.02.99

**Published:** 2016-08-28

**Authors:** Jubbin Jagan Jacob

**Affiliations:** Christian Medical College and Hospital, Ludhiana, Punjab, India

**Keywords:** Congenital hypothyroidism, neonatal screening, newborn screening

## Abstract

Neonatal screening for congenital hypothyroidism, along with eradication of iodine deficiency in large parts of the world, has made it possible to prevent the development of permanent neurological impairment due to thyroid hormone deficiency in the developing brain. The first successful screening programme was demonstrated in Canada in 1973 and since then it has been standard of care in most developed societies. In India there is no national programme for neonatal screening, and screening is only done in selected larger hospitals on newborns whose parents fund it. This review summarises the current understanding of the various strategies for newborn screening that could potentially be employed in India with resource constraints. Once a case is detected, the further evaluation and determination of etiology is summarised. Treatment and long term follow-up with levothyroxine replacement is also described in detail as per current understanding.

The human foetus is capable of producing thyroid hormones at around 20 weeks of gestation. Even in the rare event that there happens to be a defect in thyroid organogenesis or an inborn error in thyroid hormone synthesis, the developing foetal brain is protected by the trans-placental supply of maternal T_4_. Within the cerebral cortex there is up-regulation of type 2 de-iodinase activity (*Figure 1*), leading to an enhanced supply of active T_3_ to the cortex protecting the foetal brain from any significant neurological impairment.^1^ This understanding underlies the remarkable success of neonatal screening programmes for thyroid dysfunction at birth in providing a good neurological prognosis for infants born with congenital hypothyroidism (CH).

## Epidemiology of congenital hypothyroidism

The incidence of CH in unscreened populations, when the diagnosis is made clinically, is around one in 6000 births.^2^ Newborn screening was first initiated in the Canadian province of Quebec in 1972 and in over 3 years, seven cases were detected among the 47,000 newborns screened.^3^ Subsequently, incidence figures from around the globe have suggested one case of CH among 3,000–4,000 newborns screened.^4^ However in the last 3 decades there has been an increase in case detection rates with current estimates of incidence rates between 1:1,400 to 1:2,800 newborns screened.^5^ The primary reason for this increase in incidence of CH may relate to the change in screening strategies (from Free T_4_ [FT_4_] to thyroid stimulating hormone [TSH]) and to the lowering of thresholds for diagnosis. Most of the additional cases identified have milder forms of CH and the incidence rates for severe CH remain the same as in the decades preceding. Another factor in the apparent increase in CH rates is probably the change in demography in most Western populations with higher percentages of Asian patients in Western screening data. Asian newborns have higher rates of thyroid dyshormogensis.^6^ A last factor contributing to the increase in the incidence rates could be related to the improved survival of preterm infants over the last three decades.^6^

Indian data about the incidence of CH is scarce. Screening for CH among 40,000 newborns at the Wadia Hospital for Children in Mumbai revealed an incidence rate of 1 in 2,640 births.^7^ Details of incidence rates published from other parts of the country are given in *Table 1*.^7^–^10^

## Aetiology of congenital hypothyroidism

A vast majority of babies born with CH have an abnormal development of the thyroid gland. Thyroid dysgenesis (abnormal thyroid gland development) accounts for 85% of all the cases of CH and is largely isolated and non-syndromic. Thyroid ectopy, hypoplasia and athyreosis are the three types of thyroid dysgenesis. Among the three, thyroid ectopy accounts for over 60% of cases and is much more common in girls than boys. Syndromic thyroid dysgenesis accompanies monogenic disorders of thyroid transcription factors (TTF)-1, TTF-2 and PAX-8 that lead to abnormal or absent thyroid development.^11^ Details of the various permanent and transient aetiologies of CH in the newborns are given in *Table 2*.

**Figure 1:**
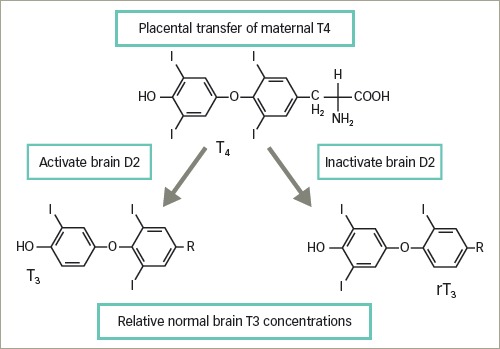
Mechanisms activated to increase availability of T3 hormones in the cerebral cortex, in the presence of low circulating T4 levels in the developing foetal brain

**Table 1:** Indian data from thyroid screening

Location	Incidence of CH	N	Method	Reference
Mumbai	1:2640	40000	Cord Blood	Desai (1997)^7^
Kolkata	1:600	1200	Cord blood	Manglik et al. (2005)^8^
Kochi	1:479	2872	72-hour heel prick	Sanghvi et al. (2008)^9^
Chandigarh	1:3400	6813	24-48 hour heel prick	Kaur et al. (2010)^10^
Vellore	1:1174	41083	Cord Blood	Personal Communication

CH = congenital hypothyroidism

Thyroid dyshormonogenesis is the term used when CH ensues because of a deficiency of one the enzymes/transporters/factors required for thyroid hormone synthesis within a structurally normal thyroid gland. Most causes of dyshormonogenesis are not syndromic, except for the well-known entity named Pendreds syndrome which is associated with sensory-neural deafness.

Transient hypothyroidism may result from maternal transmission of TSH-receptor (TSH-R) blocking antibodies, iodine excess or in the presence of maternal or foetal iodine deficiency. In un-well newborns, hypoxia, sepsis and exchange transfusions for neonatal hyperbilirubinemia may contribute to transient abnormalities of thyroid functions.

In India there appears to be a relative increase in the patients with thyroid dyshormonogenesis compared to thyroid dysgenesis. At Vellore were 41,083 newborns were screened 35 babies were detected to have CH and of them 13/35 (37%) had evidence of thyroid dyshormonogenesis as the aetiology of CH. (unpublished personal communications).

## Screening strategies for congenital hypothyroidism

Screening for CH is complicated by the dramatic changes in TSH and thyroid hormone levels at birth and in the first month after birth. The magnitude of these hormonal changes differ in a preterm infant, small for gestational age (SGA) baby and a normal term appropriate for gestational age (AGA) baby, making single measurements difficult to interpret without appropriate local cut offs. The major changes in thyroid hormones at birth include:

**Table 2:** Permanent and transient causes of congenital hypothyroidism

Primary hypothyroidism permanent	Non-syndromic	Thyroid dysgenesis (75%) Thyroid ectopy (40%) Thyroid hemi-agenesis (<1%) Athyreosis (30%) Thyroid hypoplasia (5%)
		Thyroid dyshormonogenesis are caused by autosomal recessive gene defects encoding the following proteins (20%) Thyroglobulin Thyroid peroxidase Dual oxidase 2 Dual oxidase maturation factor 2 De-halogenase Sodium/Iodide symporter
		Other rare causes Inadvertent maternal radio-iodine exposure Allo-immune thyroiditis
	Syndromic	Thyroid dysgenesis because of mutations in TTF-2 (Bamforth Lazarus syndrome) mutations present with CH. Spiky hair and cleft palate TTF-1/NKX2-1 mutations present with CH, lung maturation disorders and choreoathetosis PAX8 mutations with renal agenesis and genito-urinary abnormalities GNAS mutation with Albrights hereditary osteodystrophy
		Thyroid dyshormonogenesis Pendreds syndrome (mutation of the Pendrin gene leading to CH, deafness and goitre)
Primary hypothyroidism transient		Iodine deficiency Maternal/foetal exposure to excess iodine Transplacental transfer of TSH-R blocking antibodies from the mother Maternal and foetal iodine deficiency Perinatal acute illness Congenital liver haemangioma (consumptive hypothyroidism due to excess type 3 de-iodinases) Downs syndrome Heterozygous THOX-2 and DUOXA2 mutations

CH = congenital hypothyroidism; GNAS = guanine nucleotide binding protein, alpha subunit stimulating; NKX2-1 = NK2 homeobox 1; PAX8 = Paired Box Gene 8; TSH-R = thyroid stimulating hormone receptor; TTF = thyroid transcription factors

- A 50% increase in total T_4_ which peaks on day 7 of infancy and stabilises by day 28
- A gradual three- to four-fold increase in total T_3_ levels which reach adult levels by the end of 28 days of life
- An abrupt increase in TSH within 6 hours of delivery compared to the cord blood TSH values followed by a dramatic fall in the first seven days of life.^12^

Because of these changes the optimal time for screening infants for CH is probably a week after birth. However, by this time, babies would have usually been discharged from hospital and collection of samples from the community would entail extra expenditure and effort. This is the reason why most Western protocols suggest that screening for healthy newborns should be done between day 2 and 5 after birth prior to discharge from the hospital. For critically ill, preterm or home delivered infants, samples can be collected at the end of the first week. In India, because of the early discharge from hospital In cases of uncomplicated normal vaginal deliveries, and the difficulty in following up babies in the community, most large teaching hospitals that undertake CH screening would obtain samples from the cord blood at the time of birth. Samples collected in the first 48 hours lead to a much higher rate of false positive results.

**Figure 2:**
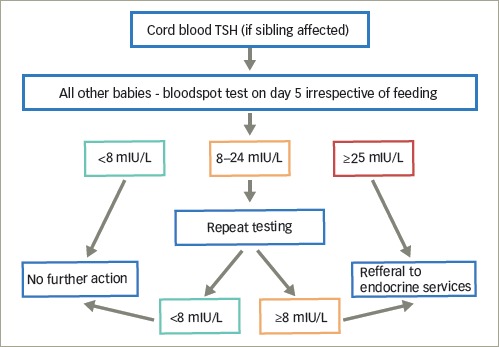
Screening protocol (primary blood spot TSH) used in the National Health Service, Scotland TSH = thyroid stimulating hormone

For the sake of convenience and simplicity the most common sample collected from babies for CH screening is a heel prick aliquot of capillary blood dried onto a filter paper. These are then transported to a central laboratory. Initial screening programmes undertook estimation of total T_4_ levels in this blood sample as the first step, followed by estimation of TSH if the total T_4_ levels fell under a pre-specified cut off. Because of improvement in the sensitivity of TSH estimation in small dried samples of blood most European, British and American programmes have now switched over to an initial TSH assay protocol. The initial TSH approach has the disadvantage of missing out on the much rarer infant with central hypothyroidism due to anterior pituitary hormone deficiencies. A recent consensus among six major paediatric organisations which included the European Society of Paediatric Endocrinology (ESPE) and the Indian Society of Pediatric and Adult Endocrinology (ISPAE), suggested that the primary priority in neonatal screening programmes should be detection of all forms of primary hypothyroidism, and TSH determination remains the most sensitive test detecting primary hypothyroidism.^13^ The different protocols for thyroid screening are described below.

### Primary TSH testing between days 2-5 using heel prick

This is by far the commonest protocol used in Western countries and the current screening test of choice in the recent guideline by major worldwide paediatric endocrine organisations.^13^ The exceptions are some areas in the United States and Netherlands. TSH is tested in a central laboratory from the blood spots dried on filter paper. The sample protocol followed by the National Health Service in Scotland is described in *Figure 2*. The disadvantages with this approach are:

- Rare patients with central hypothyroidism (Incidence 1:45000 babies) will be missed
- Isolated hypothyroxinemia
- Transient hypothyroxinemia of prematurity will not be detected and
- Patients with thyroglobulin deficiency and delayed rise in TSH will be missed

**Figure 3:**
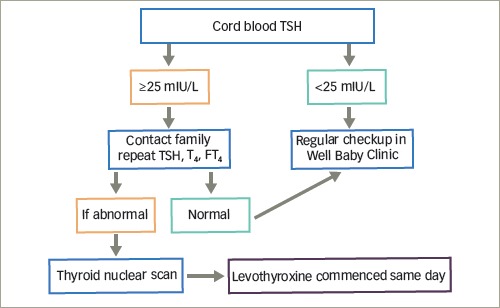
Screening protocol (cord blood TSH) used at Christian Medical College and Hospital, Vellore TSH = thyroid stimulating hormone, FT_4_ = Free T_4_

Problems with delayed TSH rise are also seen in preterm babies and very low birth weight babies. This approach usually has a recall rate of two babies for every baby with CH who is identified.^14^ Moreover when resource constraints are encountered as in India, primary TSH testing will help identify most cases of mild to severe primary hypothyroidism. A second screening should be considered in preterm, low birth weight and acutely unwell neonates.^13^ Current recommendations are to treat all patients with a capillary TSH ≥40 mIU/L after drawing a venous sample for a complete thyroid profile. If the FT_4_ on the venous blood is below the normal ranges for age, treatment should be started immediately. If the venous TSH is >20 mIU/L with FT_4_ in the normal range, treatment should still be started. In patients with normal FT_4_ and a TSH ≥6 mIU/L and <20 mIU/L further investigations should be ordered including a thyroid scanning. If patient is unable to come for a repeat testing in 2 weeks’ time treatment should be started after discussion with the parents.^13^

### TSH testing in cord blood

TSH testing in cord blood is likely to encounter lots of false alarms. However, this is the easiest sample to be obtained at the time of active delivery. A protocol followed at Christian Medical College, Vellore is described in *Figure 3*.

### Primary T_4_ (total) testing followed by TSH if required

An alternate to the above is primary total T_4_ testing followed by TSH estimation in the same sample when the measured total T_4_ is below a defined threshold (e.g., below -0.8 standard deviation [SD] of the days mean T_4_).^15^ To improve the sensitivity of total T_4_ testing, clinicians in the Netherlands also measure thyroid binding globulin in the filter paper sample when T_4_ is -1.6 SD or less. Using this method, the Netherlands group showed an incidence rate of central hypothyroidism in 1 out of 20,263 babies screened.^16^ Despite the favourable reports of this approach in Europe the first pathway of primary TSH testing is the one which is widely followed. The major disadvantages of this method are subclinical hypothyroidism will be missed, and delayed TSH rise with normal T_4_ will be missed. However, the main disadvantage of this protocol is the much larger number of patients that need to be recalled (around 12 babies) to diagnose one baby with CH.^17^

## Further evaluation of babies diagnosed with congenital hypothyroidism

### Clinical Assessment

Babies referred with abnormal screening tests should be seen promptly in specialist clinics. Clinical history would focus on symptoms of hypothyroidism in the baby (poor feeding, constipation, prolonged jaundice of newborn, hoarse cry, sleepiness and cold extremities). Birth weight and gestational age should be noted. History of thyroid disease or the use of anti-thyroid drugs in mother should be enquired about. Wellbeing of siblings and any history of co-sanguinity should be noted. Examination should focus on anthropometry (weight, head circumference and length), presence of goitre and signs of hypothyroidism (wide open anterior fontanelle, coarse features, lethargy, cold extremities, jaundice and dry skin).

Additionally, cardiac examination and hip examination should be done as there is an increased prevalence of these disorders with CH.^13,18^ Dysmorphic features should be looked for to consider syndromes associated with transient and permanent CH. These features are described in *Table 2*.

### Investigations

At least 1 ml of venous blood should be collected for thyroid function testing and an additional 1 ml should be drawn to estimate thyroglobulin. If there is a maternal family history of thyroid disease then samples should be drawn from the mother and baby for antithyroid peroxidase (TPO) antibodies and TSH-R blocking antibodies. In cases where there is parental consanguinity, DNA samples can be collected from the parents and the baby.

Some centres would routinely do a knee x-ray in babies with CH to document the bone retardation. This correlates with the degree of intra-uterine hypothyroidism and may predict future intelligent quotient (IQ) outcome and is recommended in the current guideline.^13,19^

### Diagnostic imaging

Most guidelines recommend thyroid imaging using a combination of thyroid ultrasound and isotope imaging for all babies with CH. This should be done before commencing treatment or within 5 days of commencing treatment. Isotope scanning will be indeterminate once TSH levels have suppressed <5 mIU/L. Regardless of when the imaging is conducted, it should not result in postponement of starting levothyroxine replacement therapy. In a recent review the benefits or early imaging were enumerated as:^20^

- Aids genetic counselling and in subsequently born siblings helps in early diagnosis especially if there is dyshormonogenetic CH
- Provides the parents with a visual proof of the nature of the disease (especially as many of these children are asymptomatic) and the need for life long uninterrupted levothyroxine therapy
- Gives a firm aetiological diagnosis, removes uncertainty and provides a firm basis for what is likely to be a lifelong therapy
- The precise aetiology of CH may help in guiding the levothyroxine dosing

If it is not possible to image within the specified period of time, then patients should continue treatment regardless, and investigation can be done at a later date by stopping thyroxin therapy for a period of time, after the child is at least 24 months old.

### Further investigations

Ectopic thyroid and athyreosis with undetectable serum thyroglobulin leads to straight forward confirmation of diagnosis. Normal or hypoplastic glands with reduced isotope uptake should prompt investigations for maternal antibodies (anti-TPO and TSH-R). DNA analysis for TSH-R mutations can be undertaken if available and if there are no maternal antibodies to account for the low uptake.

A large or normal sized gland with normal or increase isotope uptake is suggestive of dyshormonogenetic CH. If iodine insufficiency is suspected then urine iodine excretion should be determined in both mother and child. The remaining causes of dyshormonogenesis are inherited enzyme defects, and require mutational analysis.

## Treatment and follow up

### Treatment

Replacement with levothyroxine should be administered as soon as the diagnosis is established and should not be needlessly postponed for investigations to determine the aetiology of CH. The aim of therapy is to render the newborn euthyroid as early as possible to prevent any permanent neurological sequelae. There is a direct inverse relationship between the age of starting treatment and the IQ of the child.^21^ Levothyroxine is the drug of choice and currently only the oral formulation is available in India. In Europe liquid thyroxine formulations are available. Liquid formulations have suffered from variable bioavailability in the past and so have traditionally been avoided.^22^

Currently only oral levothyroxine is used in India. The tablets are crushed and mixed with breast milk, boiled water or formula feeds to prepare a suspension. Guidelines suggest the use of branded thyroxine preparations rather than generics.^13^ This suspension is then either placed under the cheek pad of the infant or put over the mother’s nipple for the infant to feed on. For some it might be convenient to use a syringe instead of a spoon to place the suspension under the infant’s cheek pad. It almost impossible to administer levothyroxine to newborns with an empty stomach and hence this is usually administered with or after feeds. Care is taken to avoid substances like oral iron, calcium preparations and other drugs with thyroxine as these may interfere with the absorption of the drug. Parents should be instructed to err on the side of over treatment in case they notice that the baby has vomited or regurgitated the suspension. A detailed leaflet with all instructions and basic information about levothyroxine therapy and CH is a useful tool to educate parents.

The goal of therapy is to normalise FT_4_ and TSH within a fortnight of commencing therapy. Longer time periods for correction are associated with poorer neurological outcomes.^23^ The starting doses advised are 50 μgm/day of levothyroxine for babies who weigh ≥2.5 kg and a dose of 15 μg/kg/day for babies who weigh less than 2.5 kg. In first months, thyroid functions are done weekly.^20^ The TSH levels are aimed to be normalised in about 2 weeks to <5 mIU/L. During the first couple of weeks the FT_4_ levels may be kept even above the normal reference range. After the initial normalisation of TSH, doses can be down titrated to keep the TSH between 0.5-5 mIU/L and the FT_4_ in the top quartile of the normal range.^24^ After 2 weeks the levothyroxine doses in most cases will need to down titrated to 37.5 μgm/day or even lower. The recent consensus suggests follow-up in 2 weeks after initiation of treatment and keeping the TSH within the age-specific range (which is not available in all populations) and the total T_4_ in the upper half of the age-specific range if available. Thyroid functions should be measured at least 4 hours after thyroxine dosing.^13^

After the first 3 weeks it was previously recommended that the levothyroxine dosing be based on body surface area (length [cm] X weight [kg]/3600). Tailoring the exact dose will also depend on the aetiology of CH. For patients with athyreosis a dose of 100 μgm/m^2^/ day is required. But for milder forms of CH a lower dose might provide adequate replacement.^23^

### Frequency and structure of follow up visits

After weekly visits in the first month of life, visits once every 2-4 weeks are suggested in the following six months. After 6 months of life visits can be monthly or once in 2 months till the baby is a year old. Thereafter, until 3 years of age, visits are planned every 4 months. Once the child reaches 3 years old, an annual or a 6 monthly visit is enough. In patients with concerns about poor compliance and abnormal thyroid function tests on a particular visit an early follow up visit may be more appropriate. At each visit the following minimum schedule should be followed:

- Measure anthropometry including weight, length (until age 2) and then height and head circumference until the age 3
- Calculate body surface area and adjust doses pre-emptively. Thyroid functions test help assess compliance
- Monitor development especially school performance in school going children. If there are any concerns, then a formal referral to a child psychologist should be made
- Parents should be re-educated about the need for life long treatment at every visit and once the child attains a degree of capacity and insight the child should be educated about the disease and its treatment
- A formal audiological assessment should be done once prior to entry to school^25^
- Assessment of visual processing skills in addition to visual acuity is recommended in all children with CH^13^
- Speech therapy referral should be undertaken if there is a delay in speech beyond 3 years^13^

### Auxological and neurodevelopmental outcomes in treated children

Current guidelines suggest that parents and patients should be reassured about normal growth, puberty and fertility with appropriate and adequate treatment.^13^ Children treated in the early decades of CH screening exhibited subtle neurodevelopmental and physical impairments and were also reported to have a reduced quality of life.^25^ With current practice changes and early treatment these impairments are unlikely to happen.

Most children with CH regardless of severity are likely to have neurocognition and behaviour on an average within the normal limits.^17^ However a few children with CH are likely to face clinically significant learning disability.^26,27^

## Conclusion

The overall outcomes of babies born with CH are good. Early and high dose levothyroxine with normalization of TSH within 2 weeks of therapy has improved neurological outcomes from previously. Screening protocols need to be planned as per local circumstances, but a clear plan needs to made for further evaluation and commencing early treatment with levothyroxine. In India, the logistics for universal screening are likely to be enormous and the cost required for this is not likely to be available in the near future. Most screening is done in targeted populations at individual hospitals were the parents pay for the screening. A uniform protocol to be followed across the country is also likely to be difficult considering the different patterns of health care access across the country.
